# Assessment of Physicochemical Properties and Heavy Metal Content of Floriculture Soil in Amhara Region of Northwest Ethiopia

**DOI:** 10.1155/2024/9945257

**Published:** 2024-08-19

**Authors:** Endalamaw Yihune, Solomon Addisu

**Affiliations:** ^1^ Department of Biology College of Science Bahir Dar University, Bahir Dar, Ethiopia; ^2^ Department of Natural Resources Management College of Agriculture and Environmental Science Bahir Dar University, Bahir Dar, Ethiopia

## Abstract

Floriculture is a new and rapidly expanding sector in Ethiopia that aids economic growth but has also come under blame for pollution of the surrounding soil. The purpose of this study was to assess the soil physicochemical properties and heavy metal contents in floriculture in the Amhara Region of Northwest Ethiopia. Soil samples were collected from seven different greenhouses (2ABC, 4DEF, 5ABC, 7DEF, 8ABC, 9DEF, and 11DEF), and a control soil sample was taken on the 15-cm depth from a nearby agricultural area. They were analyzed for soil physicochemical parameters and heavy metal compositions. Soil texture showed a significant difference between the soils sampled from the greenhouses and the control group. The highest average clay, silt, and sand contents were recorded in the control group, 4DEF, and 9DEF, respectively. The lower clay content was at 9 DEF, silt at 11 DEF, and sand in the control group. Clay was positively correlated to soil pH (*r* = 0.66) and TN (*r* = 0.38) but showed significant negative correlation with the sand fraction (*r* = −0.96). The average bulk density (BD) values of the soils from the greenhouses were within acceptable ranges; however, the mean BD value of 7DEF was relatively highest (1.34 g/cm^3^). There were significant (*P* < 0.05) changes in soil pH and electrical conductivity, with pH values ranging from 5.8 to 7.17 and EC from 0.08 to 1.72 mScm^−1^. Soil organic carbon, available phosphorus, total nitrogen, and carbon-to-nitrogen ratio of the soil samples from the greenhouses and the control group were significantly different. There were also significant differences in soil exchangeable aluminum and acidity between greenhouse soil samples and the control group. Soil contents of some of the heavy metals (Pb, Cd, Mn, and Cu) in the floriculture soil were above the permissible limits, while Cr, Zn, and Ni contents were below. The soil in floriculture showed low quality compared to the control group and international standards, indicating the need for improved soil quality management. This study recommends reducing agrochemical use, increasing bio-fertilizers, using botanicals, and transitioning to organic farming. Further studies are needed to assess soil microbial diversity and abundance for soil fixation.

## 1. Introduction

Floriculture is a segment of horticulture and is concerned with the cultivation of flowering and ornamental plants [1], commercializing bedding plants, cutting flowers, potted flowering plants, and noncommercial home gardening [2]. It has reached a historical maximum hub of activity and competitiveness due to the continuous development of greenhouse technology, advances in plant biotechnology, and marketability [3, 4]. In Ethiopia, floriculture is young (it began in the mid-1990s), the fast-growing industry that has grown to become the world's fourth largest flower exporter and Africa's second largest, employing over a hundred thousand people and generating foreign currency [2, 5].

Despite the floriculture industry contributes significantly to the national economy through the export of cut flowers and the creation of jobs [1, 6], it is blamed for polluting the environment and posing health risks [2]. Agrochemical residues, such as heavy metals and organic compounds, can destroy beneficial organisms in the soil [7], and it is well recognized that soil contamination can lead to water pollution if toxic chemicals leak into groundwater or contaminated runoff reaches streams, lakes, or seas [8, 9]. For instance, heavy metal inputs and contaminants such as cadmium (Cd) and lead (Pb) from phosphorus and superphosphate fertilizers can cause serious problems in water [10] and have the potential to harm nitrogen-fixing bacteria and other soil microbes [11]. Soil pollution as part of land degradation is caused by the presence of xenobiotic chemicals or other alterations in the natural soil environment [3, 12].

Heavy metals and metalloids from leaded gasoline and paints, fertilizer application on land, animal manures, sewage sludge, pesticides, coal combustion residues, and petrochemical leakage are all possible sources of pollution [13]. Heavy metals constitute of inorganic chemical hazards, and those most commonly found at contaminated sites are lead (Pb), chromium (Cr), arsenic (As), zinc (Zn), cadmium (Cd), copper (Cu), mercury (Hg), manganese (Mn), and nickel (Ni) [14]. Heavy metals are well-known environmental pollutants due to their toxicity, environmental persistence, and bioaccumulative nature [15]. Despite the limited studies conducted across counties, the practice, chemical types and usage, and dosages can vary depending on the environment, competence, and floriculture expertise and worker knowledge. Therefore, the present study aimed to evaluate the status of heavy metal contents and physicochemical properties of floriculture soil, Amhara region, Northwest Ethiopia.

### 1.1. Objectives of the Study

#### 1.1.1. General Objective

The general objective of this study was to assess the physicochemical properties and heavy metal content of floriculture soil in the Amhara Region of Northwest Ethiopia.

#### 1.1.2. Specific Objectives: The Specific Objectives Were

- To assess floriculture soil physicochemical properties
- To determine the heavy metal pollution potential of floriculture soil

## 2. Materials and Methods

### 2.1. Description of the Study Area

**Figure 1:**
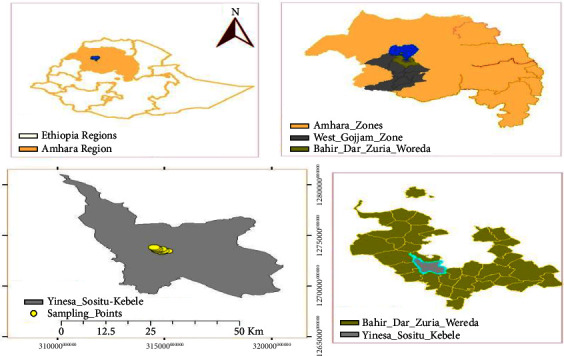
Location map of TAL floriculture (Sources: global position system (GPS) data).

The study was conducted in TAL floriculture, Bahir Dar Zuria district, Northwestern Ethiopia, 565 km northwest of Addis Ababa and 11.3 km from the regional capital city as described in Figure 1.

#### 2.1.1. Climate of the Study Area

**Figure 2:**
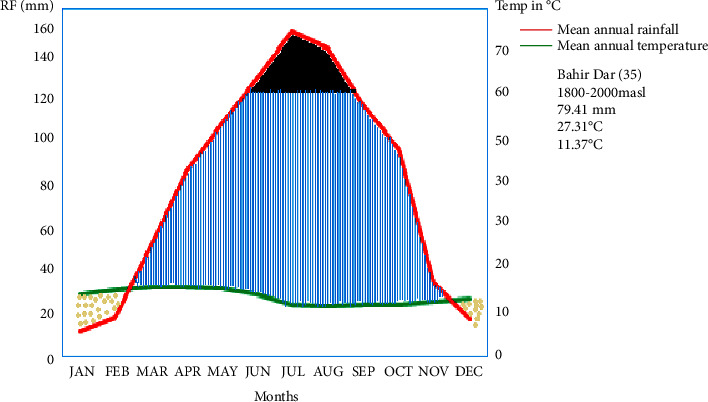
Walter climate diagram of the study area (dotted areas indicate dry periods, hatched areas humid periods, and black areas wet period).

The study area, based on 1985–2020 meteorological data, has a unimodal rainfall pattern with an average annual rainfall of 79.41 mm and a warm climatic condition with annual mean minimum and maximum temperatures of 11.37°C and 27.31°C, respectively, as described in Figure 2.

#### 2.1.2. Sampling Procedure and Sample Size Determination

In this study, soil samples were taken from seven randomly selected greenhouses (namely, 2ABC, 4DEF, 5ABC, 7DEF, 8ABC, 9DEF, and 11DEF) from a total of 14 TAL floriculture greenhouses and one soil sample from the nearby agricultural field as a control group, and thus, a total of eight soil sample sites were used for the study to assess the soil physicochemical properties and heavy metal contents. The soil samples were collected in triplicate in each of the TAL floriculture greenhouses each of them treated with unique chemical (Fe, Cu, Mg, Ca, Map, T vag, and Bilanka menda Chinese, respectively, to each of the greenhouse) and the control soil sample using soil sampler Auger. Eight soil sample pits were utilized in each greenhouse to collect samples from a depth of 0–15 cm, the sample was bulked and mixed after roots and debris were removed, and three composite samples per greenhouse were bagged and tagged in polyethylene bag for laboratory analysis. After that, the composite soil samples were air-dried, crushed, and sieved at 2 mm mesh. Furthermore, undisturbed soil samples of known volume were obtained with a sharp-edged steel cylinder (soil sample corer) forced manually for the soil bulk density determination. Moreover, the following soil property parameters were analyzed in the soil laboratory: soil texture, bulk density, soil pH (H_2_O), soil electrical conductivity (SEC), soil organic carbon (SOC), soil organic matter (SOM), total nitrogen (TN), available phosphorous (Ava P), C : N ratio, exchangeable Aluminum, exchangeable acidity, and hydrogen. GPS was used to identify the geographical locations of the soil sampling sites. This investigation is crucial for understanding soil quality and flower production rates.

### 2.2. Data Analysis

The data in soil physicochemical properties and heavy metal contents among TAL floriculture greenhouses and the control groups were subjected to a one-way analysis of variance (One-Way ANOVA) using SPSS Version 20, at *P* < 0.05 significant levels. Mean comparisons were employed using least significant difference (LSD) test at 5% levels, to assess variations in the soil physicochemical properties and heavy metal contents among floriculture greenhouses. Descriptive statistics (mean, standard error) were also used to analyze the relationship between soil property variables and interpret relevant data sources. Pearson correlation matrix analysis was done using corrplot R-package (R Core Team, V.4.1.3, 2022).

### 2.3. Soil Sample Laboratory Analysis

#### 2.3.1. Soil Physical Properties

Soil textural analysis (particle size) was analyzed following the hydrometer method after destroying OM using hydrogen peroxide and dispersion in a mixer with sodium hexametaphosphate (NaPO_3_)_6_ [16]. Bulk density was determined using the undisturbed core sampler method using a volumetric cylinder and calculated by dividing the oven-dry mass at 105°C by the volume of the core [16].

#### 2.3.2. Soil Chemical Properties

The pH of the soil was measured in water (pH (H_2_O)) potentiometrically using a digital pH meter in the supernatant suspension of 1 : 2.5 soils to water ratio using a glass-calomel combination electrode [17]. Soil electrical conductivity was determined using the standard method [18]. Total nitrogen (TN) content was measured using the Kjeldahl digestion, distillation, and titration method [17]. Soil organic carbon content was analyzed by wet combustion methods [19]. Soil organic matter was determined by using titrimetric methods, and then its contents were estimated from the organic carbon content by multiplying by 1.724. The soil's available phosphorous content was determined by measuring absorbance on a spectrophotometer following the method of extraction according to Bray II [20]. The exchangeable acidity was determined by percolating the soil samples with 1 M KCl solution and titrating them with 0.02 M NaOH. From the same extract, exchangeable Al^3+^ in the soil samples was titrated with a standard solution of 0.02 M HCl. Then, the exchangeable H^+^ was obtained by subtracting exchangeable Al^3+^ from exchangeable acidity, which is Al and H ions [21]. One gram of soil sample was weighed and transferred to a digestion vessel. Then, sixteen milliliters per chloric acid and four-milliliter hydrogen peroxide (4 : 1 ratio) was added to the Kjeldahl digester tube and heated at 180°C for three hours. Finally, the heavy metal contents (Pb, Cr, Cd, Cu, Zn, Ni, and Mn) were determined using the ICP-OES multielement analysis method [22].

## 3. Results and Discussion

### 3.1. Soil Physical Properties of TAL Floriculture

The soil samples collected from floriculture greenhouses and the control group soil sample in this investigation revealed a substantial (*P* < 0.05) variation in clay concentration. The results of the post-hoc analysis revealed that the soil samples used as a control group had the greatest average clay content (55.67 ± 1.20), followed by soil samples at 2ABC (51.0 ± 0.57), while 9DEF had the lowest average value (3.67 ± 0.88) Yihune et al. [23]. This considerable decrease in the percentage of clay in the greenhouses as compared to the control group may be related to the human disturbances that might alter parent material, such as continuous farming for flower production and extended chemical application. According to Gupta [24] and Dastgheyb Shirazi et al. [25], soil texture is an inherent soil property that may have contributed indirectly to the changes in particle size distribution. Also, there was a considerable difference in sand and silt fractions among the soil samples from the greenhouses and the control group. The highest (27.0 ± 0.57) and lowest (11.0 ± 1) values of the silt fraction were reported at the 4DEF and 11DEF greenhouses, respectively, Table 1, notwithstanding the notable fluctuation. On the other hand, the control groups had the lowest sand fraction (24.3 ± 1.20) and the greenhouse sites had the highest (9DEF, 70.3 ± 1.45). The greenhouse site 9DEFs observed sand soil content might be the result of intensive agricultural practices that expose the soil to excessive water flow and cause fine particles like clay to be lost. This finding agreed with the work of Gebeyaw [26], who stated that intensive farming causes soil compaction and degradation of soil properties including porosity. The result also showed that the soil textural classes of the study areas were sandy loam, clay loam, silt clay loam, and sand clay loam. Similar research findings revealed that continuous application of chemicals for floriculture affects the nature of the parent materials and increases the proportion of the sand fraction of the soil [27]. Sandy soils were poor for flower farming, more water to be freely drainage and unavailable for cultivation flower farming use.

**Figure 3:**
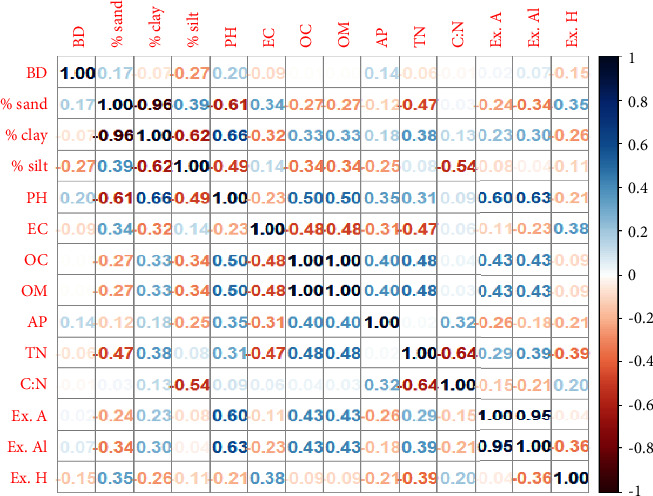
Correlation plot of the correlation matrix of the soil physicochemical properties.

As shown in Figure 3, clay had a negative and substantial association with sand (*r* = −0.96) and a positive correlation (*r* = 0.66) with the other soil physicochemical characteristics, such as pH and total nitrogen (TN). This indicated that, compared to sand and other negatively correlated features, soil clay concentration greatly raised soil pH and total nitrogen content in the soil system, with the impact being more pronounced in the control group than in the floriculture soils.

According to FAO [28], bulk density (BD) values typically range between 0.8 and 2.0 gm/cm^3^. In this study, BD showed a statistically significant difference (*P* < 0.05) between the soils in the greenhouses of the floriculture soil and the control group as described. Even though the soils in the investigated greenhouses and control group are within an acceptable range and ideal for plant growth, the greater (1.34 gm·cm^−3^) and lower (1.03 gm·cm^−3^) BD values in the greenhouses soil were obtained in 7DEF and 4DEF, respectively, as described in Table 1. The possible reason for the difference is attributed to the continuous tillage during farming activities in the greenhouse. This result is in arrangement with the research findings reported by [29]. Similarly, the low bulk density values for the soils in the floriculture greenhouses could be the result of the addition of compost and manure for floral production, which increases soil porosity and water holding capacity, in turn, reducing BD Yerima and Van Ranst [30].

### 3.2. Floriculture Soil Chemical Properties

#### 3.2.1. Soil pH, Exchangeable Acidity, and Electrical Conductivity

The results of the analysis of variance showed that there was a substantial (*P* < 0.05) variation in soil pH (H2O) between the soils in the control group and the analyzed floriculture greenhouses.  Table 2 soil sample pH for the 9DEF greenhouse was 5.8 ± 0.09, but it was higher (7.17 ± 0.03) in the control group, according to the post-hoc test. The lowest pH value observed in the greenhouse 9DEF may be attributed to ongoing application of fertilizer, pesticides, and herbicides, removal of basic cations by harvested flowers, increased microbial oxidation producing organic acids that lower soil pH by adding H ions to the soil solution. These results are in agreement with Ogumba et al. [31]. According to FAO [32], the pH of all soil samples was within the recommended range (5.5 to 7) for the availability of most essential nutrients to plant growth and development as described in Table 2, and thus, the risk of pollution in the area in terms of soil pH was low. Long-term use of pesticides and inappropriate fertilizers, such as di-ammonium phosphate (DAP) (NH_4_)_2_HPO_4_, in the soil for floral production, however, may cause toxicity and/or acidity in the soil constituents. According to the research findings, the use of certain pesticides, notably for flower production, has been proven to toxoid the soils in floriculture in Ethiopia [33].

The assessment of soil exchangeable acidity is a useful indicator of its reserve or potential acidity, particularly in the floriculture business, which uses a lot of chemicals that are thought to cause soil toxicity [1]. The investigated soils in floriculture greenhouses differed significantly (*P* < 0.05) in exchangeable acidity from the control group in this study.  Table 2 shows that the exchangeable acidity ranged from 2.83 to 4.16 cmol^+^·kg^−1^, with the greatest average value found in 2ABC greenhouses and the lowest in 9DEF greenhouses. This might be explained by the fact that regular applications of insecticides, herbicides, and inorganic fertilizers in the greenhouse leave behind acidic cations. On the other hand, the buildup of organic matter from the flower litters' leaves may be the cause of the low rate of acidity under the greenhouse of TAL floriculture. This was in line with the findings of [2].

Furthermore, Table 2 indicates a statistically significant (*P* < 0.05) variation in electrical conductivity between the soils in the greenhouse and the control group. Despite this difference, Table 2 shows that the soil EC values in the soil samples collected in the 8ABC greenhouse and the control group were the highest (1.72 ± 0.001 ms·cm^−1^) and lowest (0.08 ± 0.00 ms·cm^−1^), respectively. The continuous application of certain base-forming chemicals through fertilizer and pesticides may be the cause of the highest EC values under the 8ABC greenhouse soil. Consequently, some of the essential nutrients needed in large quantities for plant growth and development, like soil organic matter, total nitrogen, and available phosphorous, are also enhanced in the defined greenhouse soils which is similar with the finding of Obalum et al. [34]. The EC value shows the number of soluble salts in an extract and therefore provides an indicator of soil salinity. Low values of electrical conductivity obtain show that the soils are suitable for flower farming. [35] It is reported that soil with EC values less than 1 ms·cm^−1^ is suitable for plant growth.

#### 3.2.2. Soil Organic Carbon, Available Phosphorous, and Total Nitrogen

**Figure 4:**
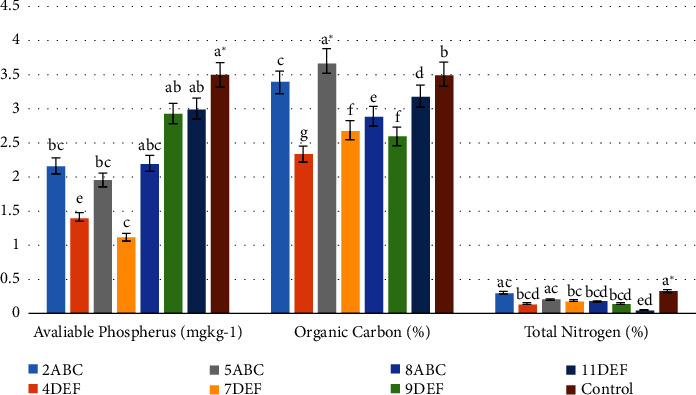
Soil organic carbon, available phosphorous, and total nitrogen values among soils of the floriculture greenhouses (Mean ± SE). Mean values within the same letters are not significantly different (*P* < 0.05), ^∗^significant at (*P* < 0.01).

Soil organic carbon, available phosphorus, and total nitrogen are the most important factors in terms of soil fertility and healthy, plant growth, crop production, soil microbial diversity, and function [36]. According to the results of the analysis of variance, there was a substantial (*P* < 0.05) variation in soil organic carbon between the soils of floriculture greenhouse and the control groups. According to Figure 4, the post-hoc test revealed that the soil organic carbon in greenhouse 2ABC was higher (3.4 ± 0.02) than in greenhouse 4DEF greenhouse soil (2.34 ± 0.02), the latter being a floriculture greenhouse. The removal of harvested flowers, ongoing use of pesticides, fertilizers, herbicides, or agrochemicals, as well as microbial oxidation that generates organic acids and decreases soil organic carbon levels, might all be contributing factors to the reduced levels of organic carbon Obalum et al. [37]. Organic carbon showed a positive correlation (*r* = 0.33) with clay, a negative correlation (*r* = 0.48) with available phosphorous, and a negative correlation (*r* = 0.48) with sand. However, TN in the analysis of variance showed that the soils in the floriculture greenhouse and the control groups are differed substantially (P0.05). According to Figure 4, the post-hoc test revealed that soil TN was lower (0.05 ± 0.01) in the soil of the 11DEF floriculture greenhouse than it was in the control group (0.32 ± 0.05). The result of this study TN of soils under the greenhouse of floriculture soil can be described as a low rate as similar with the finding of Tyopine et al. [38]. These low values of total nitrogen the area is moist that causing leaching of nitrogen despite fertilizer additions and might be related to low input of nitrogen reach organic materials such as manure, compost and unable to integrate leguminous plants on flower farm soil that fix nitrogen. The low nitrogen content likewise [39] reported that total nitrogen contents were lower in continuously farming of floriculture and use of agrochemicals.

The soil available phosphorus in the control group and floriculture greenhouse soils differed considerably (*P* < 0.05) from each other, according to the analysis of variance. According to Figure 4, the soil available phosphorus was decreased (1.12 ± 0.13) in the soil of the 7DEF floriculture greenhouse, while it was higher (3.5 ± 0.04) in the control group. This was revealed by the post-hoc test. According to Barber [40], the available phosphorus content less than 5 mg·kg^−1^ is rated low. Consequently, it was discovered that the soils of floriculture greenhouses had a low rate of available phosphorus. The nature of the parent material from which the soils are produced, the extraction of more phosphorus by flower plantations, and the leaching and fixing of iron and aluminum, which are abundant in contaminated soils, might all be the reasons for the lower reported available phosphorus content in floriculture soil that is congruent with the finding of [41].

#### 3.2.3. Soil Organic Matter and Carbon-Nitrogen Ratio (C : N)

**Figure 5:**
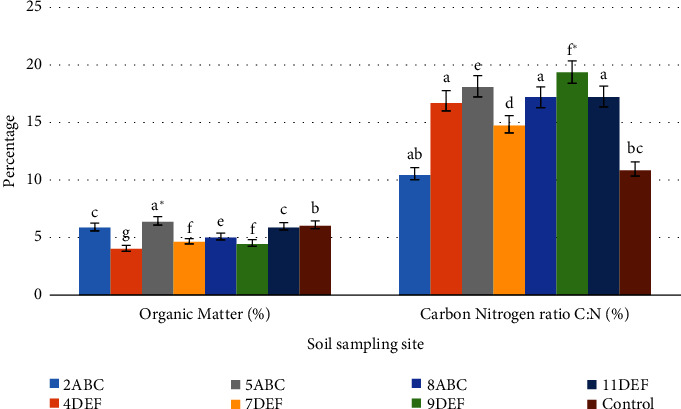
Soil organic matter and carbon-nitrogen ratio (C : N). Mean values within the same letters are not significantly different (*P* < 0.05), ^∗^significant at (*P* < 0.01).

Soil OM and C : N have an important influence on soil's physical and chemical characteristics, soil fertility status, plant nutrition, and biological activities in the soil [29]. The analysis of variance revealed that soil OM was significantly (*P* < 0.05) different among the soils in the floriculture greenhouse and the control groups. The post-hoc test showed that soil OM was greater (4.0 ± 0.38%) in the 5ABC greenhouse but lower (4.0 ± 0.38%) in the soil of the 4DEF floriculture greenhouse as described in Figure 5. This result is in agreement with [42].

The soil carbon-nitrogen ratio (C : N) in the floriculture greenhouse and control groups differed considerably (*P* < 0.05) according to the results of the analysis of variance (ANOVA). According to Figure 5, the post-hoc test revealed that the soil C : N ratio was higher (19.4 ± 2.7) in the 9DEF greenhouse but lower (10.5 ± 2) in the 2ABC floriculture greenhouse. A similar study by [43] states that high C : N ratios therefore suggest that the OM contents were not well mineralized (immobilizations were strongly preferred). High soil C : N ratios can limit soil microbial activity and reduce nitrogen mobilization, which can slow down the pace at which organic matter and nitrogen break down. On the other hand, a low soil C : N ratio may hasten the microbial breakdown of nitrogen and organic materials. This study is congruent to the findings of Ogumba et al. [31]. According to Figure 3, when OM was connected with various soil physicochemical characteristics, it was negatively correlated with sand (*r* = 0.27), silt (*r* = 0.34), EC (0.48), and clay (*r* = 0.33), pH (*r* = 0.5), available phosphorous (*r* = 0.4), and TN (*r* = 0.48). This indicated that the amount of clay in the soil raises the amount of total nitrogen and soil organic carbon in the soil system more than sand does and that these two attributes are negatively correlated. The result was more pronounced in the control group than in the soils used for floriculture.

### 3.3. Heavy Metal Contents of Floriculture

In the present study, the heavy metal content showed a significant (*P* < 0.05) difference between the soils sampled in floriculture greenhouses and the control group. The post-hoc test showed the highest average chromium (Cr) content was recorded under greenhouse 9 DEF (51.35 ± 0.09 mgkg^−1^) followed by greenhouse 7DEF (47.20 ± 0.03 mgkg^−1^), whereas the lower average value of Cr (22.13 ± 0.12 mgkg^−1^) was recorded in the control group as described in Table 3. In all collected floriculture soil samples, the concentration of Cr was recorded below the permissible limit set by CCME (100 mg·kg^−1^). This significant increment of Cr in the greenhouses compared to the control group could be associated with the result of anthropogenic activities, such as prolonged application of agro-chemicals like fertilizer, herbicides, and pesticide chemicals. Excess chromium in the soil has an unfavorable or toxic effect on plants, animals, and humans. The oxyanion chromate Cro_4_^2-^ is highly mobile and more toxic in soils [44].

In the present study, lead content showed a significant (*P* < 0.05) difference between the soils sampled in floriculture greenhouses and the control group as described. The mean total concentration of lead in the floriculture soil samples was highest in the 2ABC (9.80 ± 0.10 mg·kg^−1^) greenhouse, followed by 4DEF (9.62 ± 0.21 mg·kg^−1^), whereas the lowest average lead content was recorded in the control group (7.32 ± 0.02 mg·kg^1^). In all collected floriculture soil samples, the concentration of lead was recorded above the permissible limit set by CCME (0.66 mg·kg^−1^). This might be due to the prolonged application of agrochemicals.

**Figure 6:**
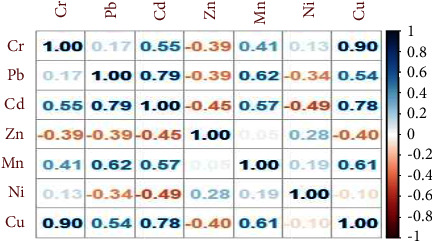
Correlation plot of the correlation matrix of the soil heavy metal content of floriculture (Source: experimental data, 2022).

According to research findings, lead is a common heavy metal pollutant that must have a negative impact on soil health in order to affect plant development [14]. In terms of correlation with other soil heavy metal contents, Cr was positively correlated with cadmium (*r* = 0.55), manganese (*r* = 0.41), and copper (*r* = 0.90) but negatively correlated with zinc (*r* = −0.39) (Figure 6).

The analysis of variance revealed that soil Cd content was significantly (*P* < 0.05) varied among soils in the TAL floriculture greenhouse as described. The post-hoc test showed that soil Cd content was higher (10.55 ± 0.03 mg·kg^−1^) in the greenhouse 5ABC but lower (5.82 ± 0.03 mg·kg^−1^) in the soil of the control group. In all the collected floriculture, soil sample concentrations of cadmium were recorded above the maximum permissible limit of CCME (1.4 mg·kg^−1^). Therefore, the high Cd value may have resulted from the frequent use of fertilizers for floriculture activities, which are the sources of Cd in the soil. The present study is in agreement with the findings of Mico Llopis et al. [45], who stated that heavy metal contaminants affect the agricultural soils.

The analysis of variance revealed that floriculture soil Zn content was significantly (*P* < 0.05) varied among the soils in the floriculture soil greenhouse. The post-hoc test showed that floriculture soil Zn content was greater (14.37 ± 0.19 mg·kg^−1^) in a control group but lower (9.43 ± 0.32 mg·kg^−1^) in the soil of 7 DEF floriculture greenhouse. In soil samples of floriculture soil, the concentration of zinc content was recorded below the permissible limit set by CCME (<50 mg·kg^−1^). This work corresponds to the findings of [46]. In terms of correlation with other soil heavy metal contents, Zn was positively correlated with Ni (*r* = 0.28) and manganese (*r* = 0.05) but negatively correlated with Cr, Pb (*r* = −0.39), Cd (*r* = −0.45), and Cu (*r* = −0.40) (Figure 6). The instrument working conditions for the determination of heavy metals in a soil sample by ICP-OES standard metal analysis equipment's are shown in Table 4.

Manganese (Mn) is one of the most common elements and essential metals for all living organisms within the acceptable range, but it exceeds the permissible limits and can be an important environmental and soil pollutant. The analysis of variance revealed that floriculture Mn content was significantly (*P* < 0.05) different among the soils in the floriculture greenhouse. The post-hoc test showed that floriculture soil Mn content was greater (482 ± 0.73 mg·kg^−1^) in the 5ABC greenhouse, followed by greenhouse 9DEF (467 ± 0.72 mg·kg^−1^) but lower (261 ± 0.13 mg·kg^−1^) in the control group of floriculture, as described in Table 3. The concentration of manganese in all floriculture soil samples was above the maximum permissible limit set by CCME (64 mg/kg). This very high concentration of Mn might be from the repetitive use of agrochemicals (fertilizer, herbicides, and pesticides) and the soil parent materials, which are a natural source of Mn in the soil. This study is similar to the report of [47].

The analysis of variance showed that floriculture Ni content was significantly (*P* < 0.05) different between the soils in the TAL floriculture greenhouse. Nickel (Ni) is a naturally occurring metal and essential for plant growth at low concentrations; however, Ni pollution increases in the soil environment due to the use of fertilizers, and chemicals have toxic effects on floral growth [46].

As described in the post-hoc test, floriculture soil Ni content was greater (41.50 ± 0.09 mg·kg^−1^) in the 9 DEF greenhouse, followed by the control group (38.83 ± 0.04 mg·kg^−1^) but lower (35.38 ± 0.20 mg·kg^−1^) in the 4 DEF of floriculture, as described in Table 3. The concentration of Ni in all floriculture soil samples was below the maximum permissible limit set by CCME (100 mg·kg^−1^). A similar work was reported by [46].

The analysis of variance showed that floriculture soil Cu content was significantly (*P* < 0.05) different between the soils in the floriculture greenhouse. The post-hoc test revealed that floriculture soil Cu content was greater (102.9 ± 0.58 mg·kg^−1^) in the 9DEF greenhouse, followed by the 2ABC greenhouse (99.3 ± 0.41 mg·kg^−1^) but lower (50.8 ± 0.39 mg·kg^−1^) in the control group of floriculture soil, as described in Table 3. The concentration of Cu in all floriculture soil samples was above the maximum permissible limit set by CCME (1.3 mg·kg^−1^). Copper (Cu) contamination of agricultural soils is a great concern due to its wide and continuous use in agriculture and horticulture as a fertilizer and fungicide [48]. A similar study was reported by [42]. In terms of correlation with other soil heavy metal contents, Cu was positively correlated to Cr (*r* = 0.90), Pb (*r* = 0.54), and cadmium (0.78) but negatively correlated with Zn (*r* = −0.40), as described in Figure 6.

## 4. Conclusion

Soil pollution from chemicals can affect soil health, microbiological organisms, and plant growth. The study found significant variations in soil physicochemical properties and heavy metal contents between greenhouse samples from floriculture and control samples. The soil samples had low bulk density, low pH values, low electrical conductivity, low total nitrogen, organic matter, high C/N ratios, and low available phosphorus. Heavy metal contents in the soils exceeded the permissible limit (Pb, Cd, Mn, and Cu), but Cr, Zn, and Ni contents were below. The major limitation with this study was the reluctance of floriculture officials to provide soil samples as well as obtaining of laboratory equipment's and chemicals. This study recommends reducing agrochemical use, increasing biofertilizers, using botanicals, and transitioning into organic farming. Further studies are needed to assess soil microbial diversity and abundance for soil fixation.

## Figures and Tables

**Table 1:** Soil texture and BD values among greenhouse in soil floriculture (mean ± SE).

Sample site	% Sand	% Clay	% Silt	Textural class	BD (gm/cm^3^)
2ABC	30.0 ± 1^f^	51.0 ± 0.57^ab^	19.0 ± 1.15^cd^	C	1.15 ± 0.05^cd^
4DEF	40.3 ± 1.2^e^	32.67 ± 0.88^c^	27.0 ± 0.57^a^∗^^	CL	1.03 ± 0.004^e^
5ABC	62.3 ± 0.88^b^	13.33 ± 0.6^e^	24.3 ± 1.2^ac^	CL	1.15 ± 0.006^cd^
7DEF	46.3 ± 1.45^d^	35.33 ± 0.88^c^	18.3 ± 0.6^d^	SCL	1.34 ± 0.007^a^∗^^
8ABC	56.0 ± 1.15^c^	21.00 ± 1.52^d^	23.0 ± 0.57^acd^	L	1.14 ± 0.005^cd^
9DEF	70.3 ± 1.45^a^∗^^	3.67 ± 0.88^f^	26.0 ± 0.57^ab^	SL	1.26 ± 0.006^ab^
11DEF	42.7 ± 0.882^e^	46.33 ± 1.2^b^	11.0 ± 1^e^	SC	1.18 ± 0.006^bc^
Control	24.3 ± 1.20^f^	55.67 ± 1.20^a^∗^^	20.0 ± 2^cd^	C	1.20 ± 0.008^bc^
LSD	10.33	7.67	12.0		0.17

Mean values within columns followed by the same letters are not a significant difference *P* < 0.05); ^∗^significant at (*P* < 0.01), but mean values with in the columns followed by different letters have significance; BD, bulk density; SE, standard error; C, clay; CL, clay loam; SC, sandy clay; SCL, sand clay loam; SL, sandy loam.

**Table 2:** Soil pH, EC, Ex. Al, Ex. H, and exchangeable acidity values between greenhouses in floriculture soil (mean ± SE).

Soil sample sites	pH (H_2_O)	EC (ms·cm^−1^)	Ex. Al (cmol^+^·kg^−1^)	Ex. H (cmol^+^·kg^−1^)	Ex. A. (cmol^+^·kg^−1^)
2ABC	6.2 ± 0.06^c^	0.45 ± 0.19^c^	2.16 ± 0.08^cd^	1.12 ± 0.04^a^	3.28 ± 0.08^b^
4DEF	6.1 ± 0.06^ef^	0.87 ± 0.001^b^	2.32 ± 0.08^bc^	1.2 ± 0.12^a^	3.52 ± 0.08^b^
5ABC	6.3 ± 0.03cd^e^	0.44 ± 0.001^c^	2.9 ± 0.13^a^∗^^	1.25 ± 0.09^a^	4.16 ± 0.08^a^∗^^
7DEF	6.5 ± 0.03^bc^	0.83 ± 0.001^b^	2.8 ± 0.05^ab^	1.17 ± 0.07^a^	3.97 ± 0.12^a^
8ABC	6.4 ± 0.06^bcd^	1.72 ± 0.001^a^∗^^	2.24 ± 0.16^c^	1.3 ± 0.09^a^	3.5 ± 0.12^b^
9DEF	5.8 ± 0.089^f^	0.54 ± 0.001^bc^	1.65 ± 0.12^d^	1.17 ± 0.07^a^	2.83 ± 0.16^c^
11DEF	6.61 ± 0.05^b^	0.70 ± 0.000^bc^	2.1 ± 0.07^cd^	1.3 ± 0.07^a^	3.4 ± 0.12^b^
Control	7.17 ± 0.03^a^∗^^	0.08 ± 0.000^d^	2.9 ± 0.12^a^∗^^	1.04 ± 0.04^a^	3.94 ± 0.12^a^
LSD	0.27	1.8	1.1	NS	0.56

Mean values within columns followed by the same letters are not a significant difference (*P* < 0.05), ^∗^significant at (*P* < 0.01), but different letters have significance difference. SE = standard error of the mean; EC = electrical conductivity; Ex. Al = exchangeable aluminum; Ex. A = exchangeable acidity.

**Table 3:** Heavy metal content of soils in the study area (mean ± SE) compared with the recommended permissible limit of agricultural soil (CCME, 2007).

Soil sample	Cr (mg·kg^−1^)	Pb (mg·kg^−1^)	Cd (mg·kg^−1^)	Zn (mg·kg^−1^)	Mn (mg·kg^−1^)	Ni (mg·kg^−1^)	Cu (mg·kg^−1^)
2ABC	40.60 ± 0.10^f^	9.80 ± 0.10^a^∗^^	9.55 ± 0.06^c^	12.18 ± 0.04^c^	441 ± 0.80^c^	37.90 ± 0.25^d^	99.3 ± 0.41^b^
4DEF	38.77 ± 0.12^g^	9.62 ± 0.21^a^	10.03 ± 0.13^b^	10.07 ± 0.03^e^	380 ± 1.36^d^	35.38 ± 0.20^f^	97.9 ± 0.32^c^
5ABC	42.97 ± 0.02^e^	8.92 ± 0.04^b^	10.55 ± 0.03^a^∗^^	14.12 ± 0.02^b^	482 ± 0.73^a^∗^^	36.77 ± 0.11^e^	98.4 ± 0.45^c^
7DEF	47.20 ± 0.03^b^	7.87 ± 0.12^d^	8.87 ± 0.04^d^	9.43 ± 0.32^g^	294 ± 0.04^f^	36.48 ± 0.07^e^	93.8 ± 0.47^d^
8ABC	46.67 ± 0.03^c^	8.53 ± 0.03^c^	9.68 ± 0.03^c^	11.45 ± 0.03^d^	295 ± 0.03^f^	37.63 ± 0.08^d^	97.2 ± 0.40^c^
9DEF	51.35 ± 0.09^a^∗^^	8.45 ± 0.08^c^	8.55 ± 0.06^e^	11.33 ± 0.02^d^	467 ± 0.72^b^	41.50 ± 0.09^a^∗^^	102.9 ± 0.58^a^∗^^
11DEF	45.93 ± 0.15^d^	7.37 ± 0.02^e^	7.47 ± 0.02^f^	14.78 ± 0.06^a^∗^^	341 ± 0.27^e^	38.37 ± 0.04^c^	96.6 ± 0.23^c^
Control	22.13 ± 0.12^h^	7.32 ± 0.02^e^	5.82 ± 0.03^g^	14.37 ± 0.19^a^	261 ± 0.13^g^	38.83 ± 0.04^b^	50.8 ± 0.39^e^
CCME	100	0.1	1.4	<50	64	100	5–60
LSD	0.73	0.66	0.48	0.41	14.9	0.46	1.3

Mean values within columns followed by the same letters are not a significant difference (*P* < 0.05), ^∗^significant at (*P* < 0.01), but different letters within the column have significant difference.

**Table 4:** The instrument working conditions for the determination of heavy metals in a soil sample by ICP-OES standard metal analysis equipment.

Heavy metal	Wavelength	Concentration of standards (mg·L^−1^)	Regression equations	CD (*R*^2^)
Cr	267.716	0.05, 1.05, 2.05, 3.05, 4.05, 5.05	*Y* = 1436000*x* − 8167.0	0.999969
Pb	220.353	0.05, 1.05, 2.05, 3.05, 4.05, 5.05	*Y* = 76940*x* − 730.4	0.999844
Cd	228.802	0.05, 1.05, 2.05, 3.05, 4.05, 5.05	*Y* = 762000*x* − 9081.6	0.999860
Zn	206.200	0.05, 1.05, 2.05, 3.05, 4.05, 5.05	*Y* = 362400*x* − 1474.7	0.998982
Mn	257.610	0.05, 1.05, 2.05, 3.05, 4.05, 5.05	*Y* = 10430000*x* − 305069.9	0.999722
Ni	231.604	0.05, 1.05, 2.05, 3.05, 4.05, 5.05	*Y* = 485000*x* − 1262.0	0.999976
Cu	327.393	0.05, 1.05, 2.05, 3.05, 4.05, 5.05	*Y* = 865100 + 25848.4	0.999604

CD = coefficient of determination.

## Data Availability

The authors volunteer to provide all data used upon a justifiable request to the corresponding author.
